# The chromosomal genome sequence of the buttercup lucine, *Anodontia alba* Link, 1807 (Lucinida: Lucinidae) and its associated microbial metagenome sequences

**DOI:** 10.12688/wellcomeopenres.25920.1

**Published:** 2026-02-17

**Authors:** Diana Chin, Barbara Campbell, Jillian Petersen, Shen Jean Lim, Graeme Oatley, Elizabeth Sinclair, Eerik Aunin, Noah Gettle, Camilla Santos, Michael Paulini, Haoyu Niu, Victoria McKenna, Rebecca O’Brien, Wellcome Sanger Institute Tree of Life Management, Samples and Laboratory team, Wellcome Sanger Institute Scientific Operations: Sequencing Operations, Wellcome Sanger Institute Tree of Life Core Informatics team, EBI Aquatic Symbiosis Genomics Data Portal Team, Aquatic Symbiosis Genomics Project Leadership

**Affiliations:** 1 Marine Science Center, Northeastern University, Nahant, MA, USA; 2 Department of Biological Sciences, Clemson University, Clemson, SC, USA; 3 Centre for Microbiology and Environmental Systems Science, University of Vienna, Vienna, Austria; 4 College of Marine Science, University of South Florida, St. Petersburg, FL, USA; 5 Tree of Life Programme, Wellcome Sanger Institute, Hinxton, England, UK

**Keywords:** Anodontia alba; buttercup lucine; genome sequence; chromosomal; Lucinida; microbial metagenome assembly

## Abstract

We present a genome assembly from an individual *Anodontia alba* (buttercup lucine; Mollusca; Bivalvia; Lucinida; Lucinidae). The genome sequence has a total length of 1 862.85 megabases. Most of the assembly (99.28%) is scaffolded into 18 chromosomal pseudomolecules. The mitochondrial genome has also been assembled, with a length of 18.48 kilobases. Gene annotation of this assembly by Ensembl identified 12 083 protein-coding genes. From the metagenome data, we recovered four bins, of which three were high-quality MAGs.

## Species taxonomy

Eukaryota; Opisthokonta; Metazoa; Eumetazoa; Bilateria; Protostomia; Spiralia; Lophotrochozoa; Mollusca; Bivalvia; Autobranchia; Heteroconchia; Euheterodonta; Imparidentia; Lucinida; Lucinoidea; Lucinidae; *Anodontia*; *Anodontia alba* Link, 1807 (NCBI:txid244458)

## Background

*Anodontia alba*, also known as the buttercup lucine, was first described by Link in 1807 ( Link, 1807). It belongs to the species-rich and widely distributed family of chemosymbiotic marine bivalves called Lucinidae. Lucinidae have a fossil record dating back to the Silurian ( Liljedahl, 1992), and fossils similar to extant *Anodontia* have been reported ( Taylor & Glover, 2005). *Anodontia alba* is placed within the morphologically diverse Leucosphaerinae subfamily ( Taylor *et al.*, 2016). All extant Lucinidae species house chemosynthetic intracellular bacteria within their gill epithelial cells ( Taylor & Glover, 2021). Known lucinid symbionts fix inorganic carbon using the energy gained from oxidising reduced sulfur compounds. This carbon is a source of nutrition for the symbionts and for their hosts. The endosymbionts can also significantly reduce the amount of sulfide in the environments they colonise, including root zones of seagrasses and seagrass sediments to make the environment less toxic ( Chin *et al.*, 2021; Reynolds *et al.*, 2007; van der Heide *et al.*, 2012). In addition to their role in nutrient cycling, lucinids are prey for commercially important species such as lobsters ( Higgs *et al.*, 2016).

*Anodontia alba* is typically found in coastal seagrass beds and is distributed in the western Atlantic, including Florida ( Lim *et al.*, 2019), Guadeloupe ( Taylor *et al.*, 2016), southern Gulf of Mexico ( Suárez-Mozo *et al.*, 2023), and as far south as Panama in the Caribbean Sea ( Osvatić *et al.*, 2021). It can grow up to 6 cm in size, and its shell has a white, smooth, sub-spherical, and toothless appearance ( Taylor *et al.*, 2016). Like many lucinid symbionts found in seagrass sediments ( König *et al.*, 2016; Morel-Letelier *et al.*, 2024; Osvatić *et al.*, 2023; Petersen *et al.*, 2016), the *Anodontia alba* symbiont from Panama has the genetic capability to fix nitrogen in addition to carbon ( Osvatić *et al.*, 2021).

Chemosynthetic symbiosis evolved several times independently in diverse animal groups ( Sogin *et al.*, 2021), and has resulted in major behavioural, physiological, and morphological adaptations in lucinids ( Taylor & Glover, 2021), which are presumably genetically encoded. However, although genome sequences are available for several other bivalve and non-bivalve hosts of chemosynthetic symbionts, few Lucinidae genomes have been sequenced ( Guo *et al.*, 2023; e.g. Sun *et al.*, 2017). Genomes of *Lucinisca nassula* ( Anderson *et al.*, 2025), *Ctena decussata* ( Wilkins *et al.*, 2025) and *Indoaustriella scarlatoi* ( Guo *et al.*, 2025) have recently been published. The genome of *A. alba* presented here will enable a new understanding of how chemosymbiosis has shaped the evolution of lucinid bivalves and allow us to identify shared or unique genomic features across animal groups that are relevant to symbiosis and/or chemosynthetic symbiosis.

## Methods

### Sample acquisition

**Figure 1.**
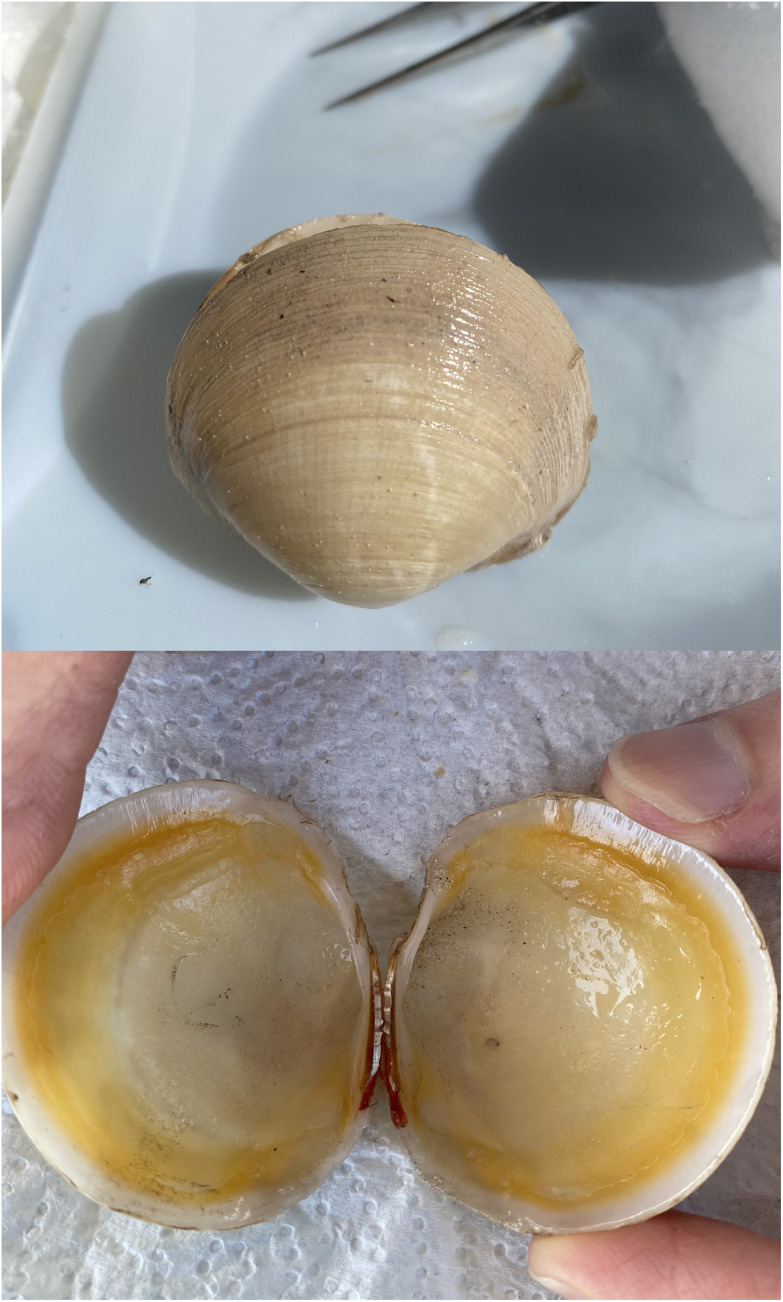
Photographs of the *Anodontia alba* (xbAnoAlba1) specimen used for genome sequencing.

An *Anodontia alba* specimen (specimen ID AG1/VIEM1130001, ToLID xbAnoAlba1; Figure 1) was collected from Sammy Creek Landing, FL, USA on 2022-02-06, where this species was previously found co-existing with at least five other lucinid species ( Lim *et al.*, 2019). The specimen was taken from its habitat of seagrass sediments by Barbara Campbell (Clemson University) and Shen Jean Lim (University of South Florida) using a shovel and sieve. The specimen was identified by Diana Chin (Northeastern University) based on gross morphology. Dissected tissues were placed immediately in a microcentrifuge tube and flash-frozen in a mixture of dry ice and ethanol. The same specimen was used for RNA sequencing.

### Nucleic acid extraction

Protocols for high molecular weight (HMW) DNA extraction developed at the Wellcome Sanger Institute (WSI) Tree of Life Core Laboratory are available on protocols.io ( Howard *et al.*, 2025). The xbAnoAlba1 sample was weighed and triaged to determine the appropriate extraction protocol. Gill tissue was homogenised by powermashing using a PowerMasher II tissue disruptor. HMW DNA was extracted using the Manual Tissue Nanobind protocol. DNA was sheared into an average fragment size of 12–20 kb following the Megaruptor®3 for LI PacBio protocol. Sheared DNA was purified by automated SPRI (solid-phase reversible immobilisation). The concentration of the sheared and purified DNA was assessed using a Nanodrop spectrophotometer and Qubit Fluorometer using the Qubit dsDNA High Sensitivity Assay kit. Fragment size distribution was evaluated by running the sample on the Femto Pulse system. For this sample, the final post-shearing DNA had a Qubit concentration of 159.0 ng/μL and a yield of 11 925.00 ng. The Femto Pulse Genomic Quality Number (GQN) was 5.40 (threshold 10 kb).

RNA was also extracted from tissue of xbAnoAlba1 in the Tree of Life Laboratory at the WSI using the RNA Extraction: Automated MagMax™ *mir*Vana protocol. The RNA concentration was assessed using a Nanodrop spectrophotometer and a Qubit Fluorometer using the Qubit RNA Broad-Range Assay kit. Analysis of the integrity of the RNA was done using the Agilent RNA 6000 Pico Kit and Eukaryotic Total RNA assay.

### PacBio HiFi library preparation and sequencing

Library preparation and sequencing were performed at the WSI Scientific Operations core. Libraries were prepared using the SMRTbell Prep Kit 3.0 (Pacific Biosciences, California, USA), following the manufacturer’s instructions. The kit includes reagents for end repair/A-tailing, adapter ligation, post-ligation SMRTbell bead clean-up, and nuclease treatment. Size selection and clean-up were performed using diluted AMPure PB beads (Pacific Biosciences). DNA concentration was quantified using a Qubit Fluorometer v4.0 (ThermoFisher Scientific) and the Qubit 1X dsDNA HS assay kit. Final library fragment size was assessed with the Agilent Femto Pulse Automated Pulsed Field CE Instrument (Agilent Technologies) using the gDNA 55 kb BAC analysis kit.

The sample was sequenced using the Sequel IIe system (Pacific Biosciences, California, USA). The concentration of the library loaded onto the Sequel IIe was in the range 40–135 pM. The SMRT link software, a PacBio web-based end-to-end workflow manager, was used to set-up and monitor the run, and to perform primary and secondary analysis of the data upon completion.

### Hi-C

***Sample preparation and crosslinking***

The Hi-C sample was prepared from 20–50 mg of frozen gill tissue from the xbAnoAlba1 sample using the Arima-HiC v2 kit (Arima Genomics). Following the manufacturer’s instructions, tissue was fixed and DNA crosslinked using TC buffer to a final formaldehyde concentration of 2%. The tissue was homogenised using the Diagnocine Power Masher-II. Crosslinked DNA was digested with a restriction enzyme master mix, biotinylated, and ligated. Clean-up was performed with SPRISelect beads before library preparation. DNA concentration was measured with the Qubit Fluorometer (Thermo Fisher Scientific) and Qubit HS Assay Kit. The biotinylation percentage was estimated using the Arima-HiC v2 QC beads.

***Hi-C library preparation and sequencing***

Biotinylated DNA constructs were fragmented using a Covaris E220 sonicator and size selected to 400–600 bp using SPRISelect beads. DNA was enriched with Arima-HiC v2 kit Enrichment beads. End repair, A-tailing, and adapter ligation were carried out with the NEBNext Ultra II DNA Library Prep Kit (New England Biolabs), following a modified protocol where library preparation occurs while DNA remains bound to the Enrichment beads. Library amplification was performed using KAPA HiFi HotStart mix and a custom Unique Dual Index (UDI) barcode set (Integrated DNA Technologies). Depending on sample concentration and biotinylation percentage determined at the crosslinking stage, libraries were amplified with 10–16 PCR cycles. Post-PCR clean-up was performed with SPRISelect beads. Libraries were quantified using the AccuClear Ultra High Sensitivity dsDNA Standards Assay Kit (Biotium) and a FLUOstar Omega plate reader (BMG Labtech).

Prior to sequencing, libraries were normalised to 10 ng/μL. Normalised libraries were quantified again to create equimolar and/or weighted 2.8 nM pools. Pool concentrations were checked using the Agilent 4200 TapeStation (Agilent) with High Sensitivity D500 reagents before sequencing. Sequencing was performed using paired-end 150 bp reads on the Illumina NovaSeq 6000.

### RNA library preparation and sequencing

Libraries were prepared using the NEBNext ^®^ Ultra™ II Directional RNA Library Prep Kit for Illumina (New England Biolabs), following the manufacturer’s instructions. Poly(A) mRNA in the total RNA solution was isolated using oligo(dT) beads, converted to cDNA, and uniquely indexed; 14 PCR cycles were performed. Libraries were size-selected to produce fragments between 100–300 bp. Libraries were quantified, normalised, pooled to a final concentration of 2.8 nM, and diluted to 150 pM for loading. Sequencing was carried out on the Illumina NovaSeq 6000, generating paired-end reads.

### Genome assembly

Prior to assembly of the PacBio HiFi reads, a database of *k*-mer counts ( *k* = 31) was generated from the filtered reads using FastK. GenomeScope2 ( Ranallo-Benavidez *et al.*, 2020) was used to analyse the *k*-mer frequency distributions, providing estimates of genome size, heterozygosity, and repeat content.

The HiFi reads were assembled using Hifiasm ( Cheng *et al.*, 2021) with the --primary option. Haplotypic duplications were identified and removed using purge_dups ( Guan *et al.*, 2020). The Hi-C reads ( Rao *et al.*, 2014) were mapped to the primary contigs using bwa-mem2 ( Vasimuddin *et al.*, 2019), and the contigs were scaffolded in YaHS ( Zhou *et al.*, 2023) with the --break option for handling potential misassemblies. The scaffolded assemblies were evaluated using Gfastats ( Formenti *et al.*, 2022), BUSCO ( Manni *et al.*, 2021) and MERQURY.FK ( Rhie *et al.*, 2020).

The mitochondrial genome was assembled using OATK ( Zhou *et al.*, 2025).

### Assembly curation

The assembly was decontaminated using the Assembly Screen for Cobionts and Contaminants ( ASCC) pipeline. TreeVal was used to generate the flat files and maps for use in curation. Manual curation was conducted primarily in PretextView and HiGlass ( Kerpedjiev *et al.*, 2018). Scaffolds were visually inspected and corrected as described by Howe *et al*. (2021). Manual corrections included 53 breaks, 61 joins, and removal of 59 haplotypic duplications, which reduced the scaffold count by 18.8%, increased the scaffold N50 by 4.2%, and reduced the total assembly length by 1.0%. The curation process is described at https://gitlab.com/wtsi-grit/rapid-curation. PretextSnapshot was used to generate a Hi-C contact map of the final assembly.

### Assembly quality assessment

The Merqury.FK tool ( Rhie *et al.*, 2020) was run in a Singularity container ( Kurtzer *et al.*, 2017) to evaluate *k*-mer completeness and assembly quality for the primary and alternate haplotypes using the *k*-mer databases ( *k* = 31) computed prior to genome assembly. The analysis outputs included assembly QV scores and completeness statistics.

The genome was analysed using the BlobToolKit pipeline, a Nextflow implementation of the earlier Snakemake version ( Challis *et al.*, 2020). The pipeline aligns PacBio reads using minimap2 ( Li, 2018) and SAMtools ( Danecek *et al.*, 2021) to generate coverage tracks. It runs BUSCO ( Manni *et al.*, 2021) using lineages identified from the NCBI Taxonomy ( Schoch *et al.*, 2020). For the three domain-level lineages, BUSCO genes are aligned to the UniProt Reference Proteomes database ( Bateman *et al.*, 2023) using DIAMOND blastp ( Buchfink *et al.*, 2021). The genome is divided into chunks based on the density of BUSCO genes from the closest taxonomic lineage, and each chunk is aligned to the UniProt Reference Proteomes database with DIAMOND blastx. Sequences without hits are chunked using seqtk and aligned to the NT database with blastn ( Altschul *et al.*, 1990). The BlobToolKit suite consolidates all outputs into a blobdir for visualisation. The BlobToolKit pipeline was developed using nf-core tooling ( Ewels *et al.*, 2020) and MultiQC ( Ewels *et al.*, 2016), with containerisation through Docker ( Merkel, 2014) and Singularity ( Kurtzer *et al.*, 2017).

## Metagenome assembly

The metagenome assembly was generated using MetaMDBG ( Benoit *et al.*, 2024) and binned usingMetaBAT2 ( Kang *et al.*, 2019). The resulting bin sets of each binning algorithm were optimised and refined using DAS Tool ( Sieber *et al.*, 2018). PROKKA ( Seemann, 2014) was used to identify tRNAs and rRNAs in each bin, CheckM ( Parks *et al.*, 2015) (checkM_DB release 2015-01-16) was used to assess bin completeness/contamination, and GTDB-Tk ( Chaumeil *et al.*, 2022) (GTDB release 214) was used to taxonomically classify bins. Taxonomic replicate bins were identified using dRep ( Olm *et al.*, 2017) with default settings (95% ANI threshold). All bins were assessed for quality and categorised as metagenome-assembled genomes (MAGs) if they met the following criteria: contamination ≤ 5%, presence of 5S, 16S, and 23S rRNA genes, at least 18 unique tRNAs, and either ≥ 90% completeness or ≥ 50% completeness with fully circularised chromosomes ( Bowers *et al.*, 2017). Bins that did not meet these thresholds, or were identified as taxonomic replicates of MAGs, were retained as ‘binned metagenomes’ provided they had ≥ 50% completeness and ≤ 10% contamination.

## Genome sequence report

### Sequence data

**Figure 2.**
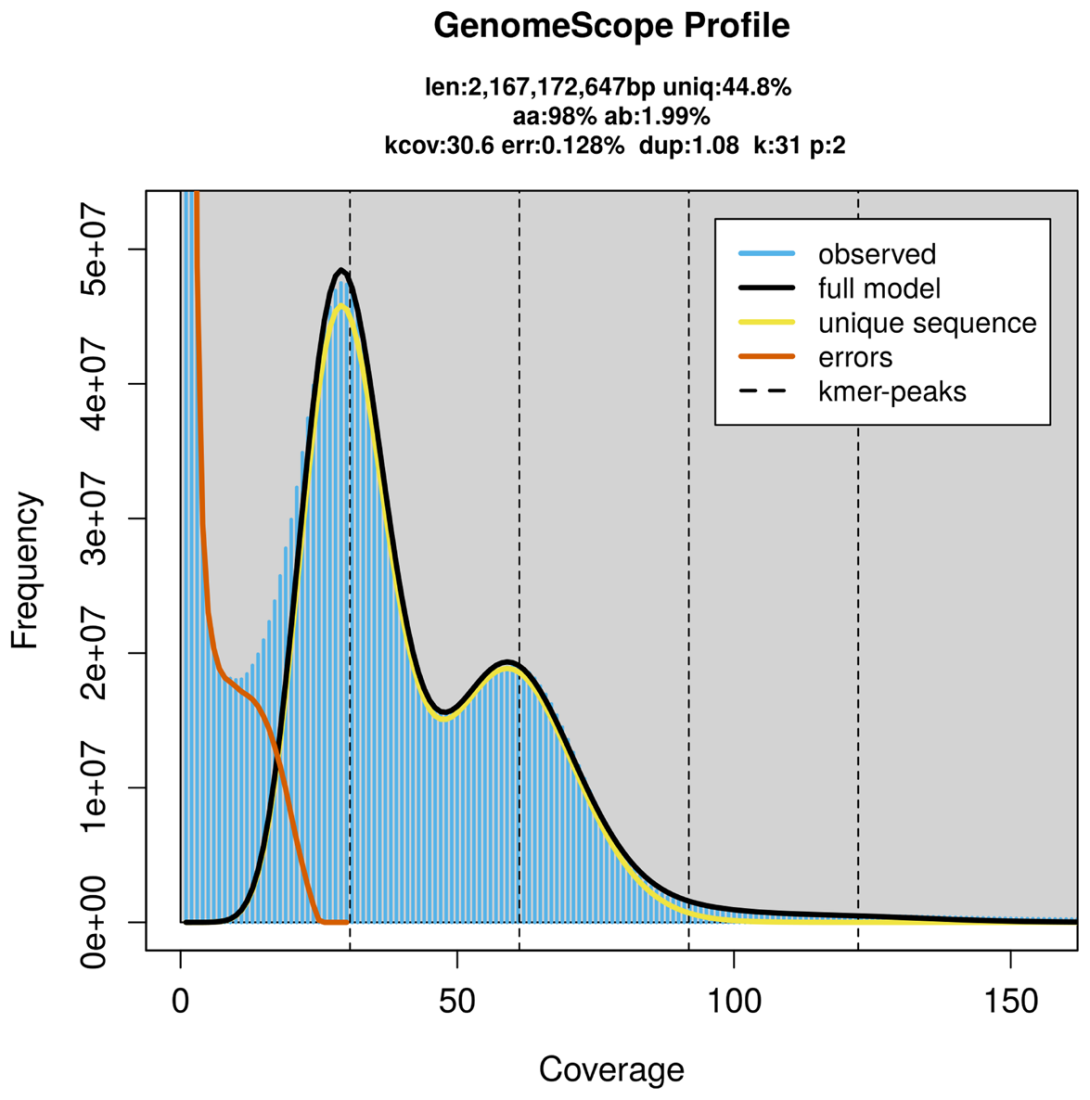
Frequency distribution of *k*-mers generated using GenomeScope2. The plot shows observed and modelled *k*-mer spectra, providing estimates of genome size, heterozygosity, and repeat content based on unassembled sequencing reads.

PacBio sequencing of the *Anodontia alba* specimen generated 159.31 Gb (gigabases) from 22.06 million reads, which were used to assemble the genome. GenomeScope2.0 analysis estimated the haploid genome size at 2 171.11 Mb, with a heterozygosity of 1.99% and repeat content of 55.18% ( Figure 2). These estimates guided expectations for the assembly. Based on the estimated genome size, the sequencing data provided approximately 50× coverage. Hi-C sequencing produced 199.50 Gb from 1 321.21 million reads, which were used to scaffold the assembly. RNA sequencing data were also generated and are available in public sequence repositories. Table 1 summarises the specimen and sequencing details.

**Table 1.** Specimen and sequencing data for BioProject PRJEB73659.

Platform	PacBio HiFi	Hi-C	RNA-seq
**ToLID**	xbAnoAlba1	xbAnoAlba1	xbAnoAlba1
**Specimen ID**	VIEM1130001	VIEM1130001	VIEM1130001
**BioSample (source individual)**	SAMEA110043978	SAMEA110043978	SAMEA110043978
**BioSample (tissue)**	SAMEA14431334	SAMEA14431338	SAMEA14431343
**Tissue**	gill	gill	**other somatic** **animal tissue**
**Instrument**	Sequel IIe	Illumina NovaSeq 6000	Illumina NovaSeq 6000
**Run accessions**	ERR12736885; ERR12736886; ERR12736882; ERR12736883; ERR12736884	ERR12737279	ERR12737280
**Read count total**	22.06 million	1 321.21 million	59.40 million
**Base count total**	159.31 Gb	199.50 Gb	8.97 Gb

### Assembly statistics

The primary haplotype was assembled, and contigs corresponding to an alternate haplotype were also deposited in INSDC databases. The final assembly has a total length of 1 862.85 Mb in 336 scaffolds, with 1 628 gaps, and a scaffold N50 of 114.1 Mb ( Table 2).

**Table 2.** Genome assembly statistics.

**Assembly name**	xbAnoAlba1.1
**Assembly accession**	GCA_964016985.1
**Alternate haplotype accession**	GCA_964016975.1
**Assembly level**	chromosome
**Span (Mb)**	1 862.85
**Number of chromosomes**	18
**Number of contigs**	1 964
**Contig N50**	1.84 Mb
**Number of scaffolds**	336
**Scaffold N50**	114.1 Mb
**Organelles**	Mitochondrion: 18.48 kb

**Figure 3.**
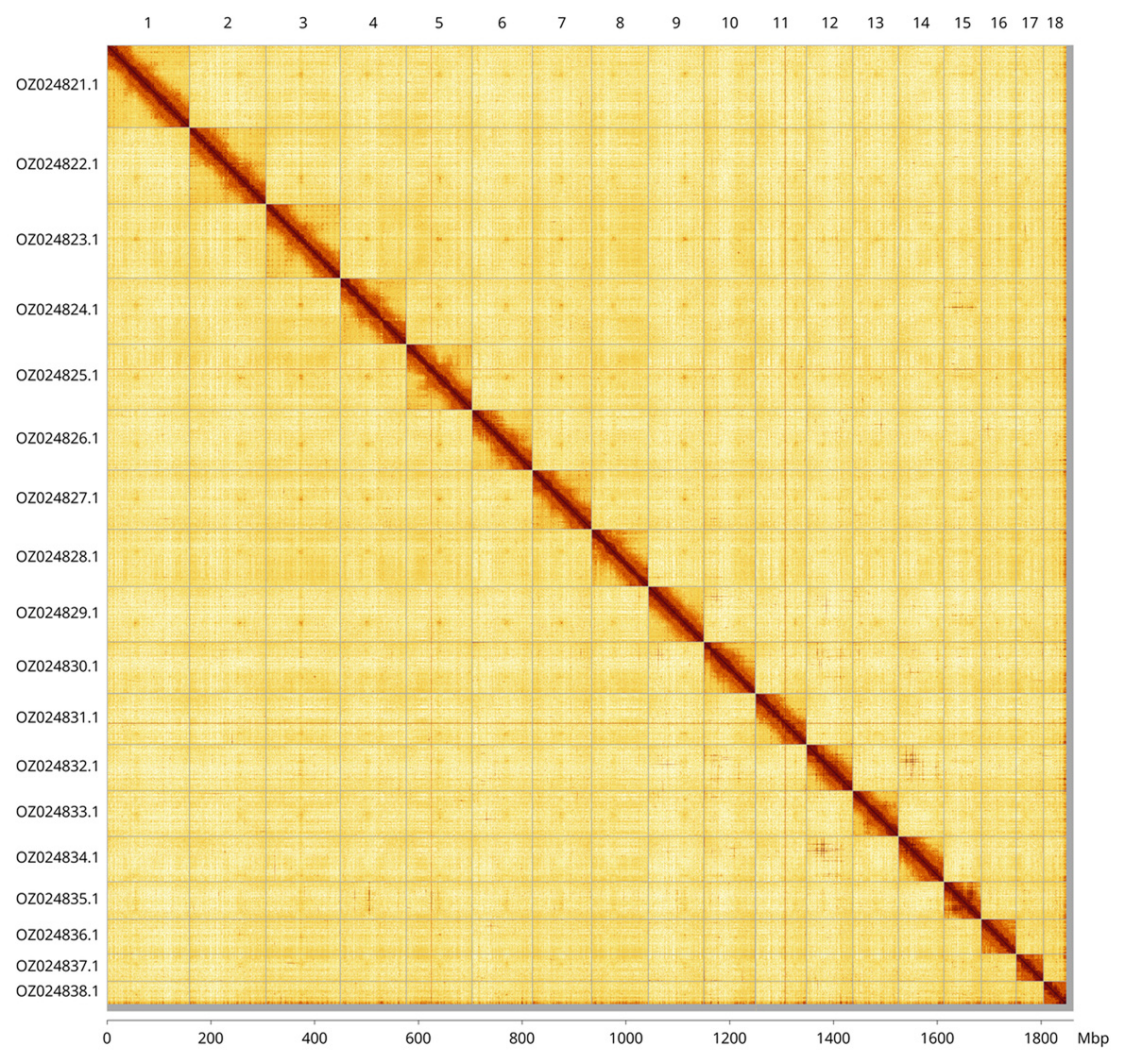
Hi-C contact map of the *Anodontia alba* genome assembly. Assembled chromosomes are shown in order of size and labelled along the axes, with a megabase scale shown below. The plot was generated using PretextSnapshot.

Most of the assembly sequence (99.28%) was assigned to 18 chromosomal-level scaffolds. These chromosome-level scaffolds, confirmed by Hi-C data, are named according to size ( Figure 3; Table 3).

**Table 3.** Chromosomal pseudomolecules in the primary genome assembly of *Anodontia alba* xbAnoAlba1.

INSDC accession	Molecule	Length (Mb)	GC%
OZ024821.1	1	158.84	37
OZ024822.1	2	147.77	37
OZ024823.1	3	143.40	37
OZ024824.1	4	126.89	37
OZ024825.1	5	126.84	37
OZ024826.1	6	116.18	37.50
OZ024827.1	7	114.10	37
OZ024828.1	8	109.66	37
OZ024829.1	9	106.94	37.50
OZ024830.1	10	99.37	37
OZ024831.1	11	98.49	37
OZ024832.1	12	88.72	37.50
OZ024833.1	13	88.13	37.50
OZ024834.1	14	87.86	37
OZ024835.1	15	71.63	37.50
OZ024836.1	16	67.47	37.50
OZ024837.1	17	52.73	37
OZ024838.1	18	44.36	37.50

The mitochondrial genome was also assembled (length 18.48 kb, OZ024839.1). This sequence is included as a contig in the multifasta file of the genome submission and as a standalone record.

### Assembly quality metrics

**Figure 4.**
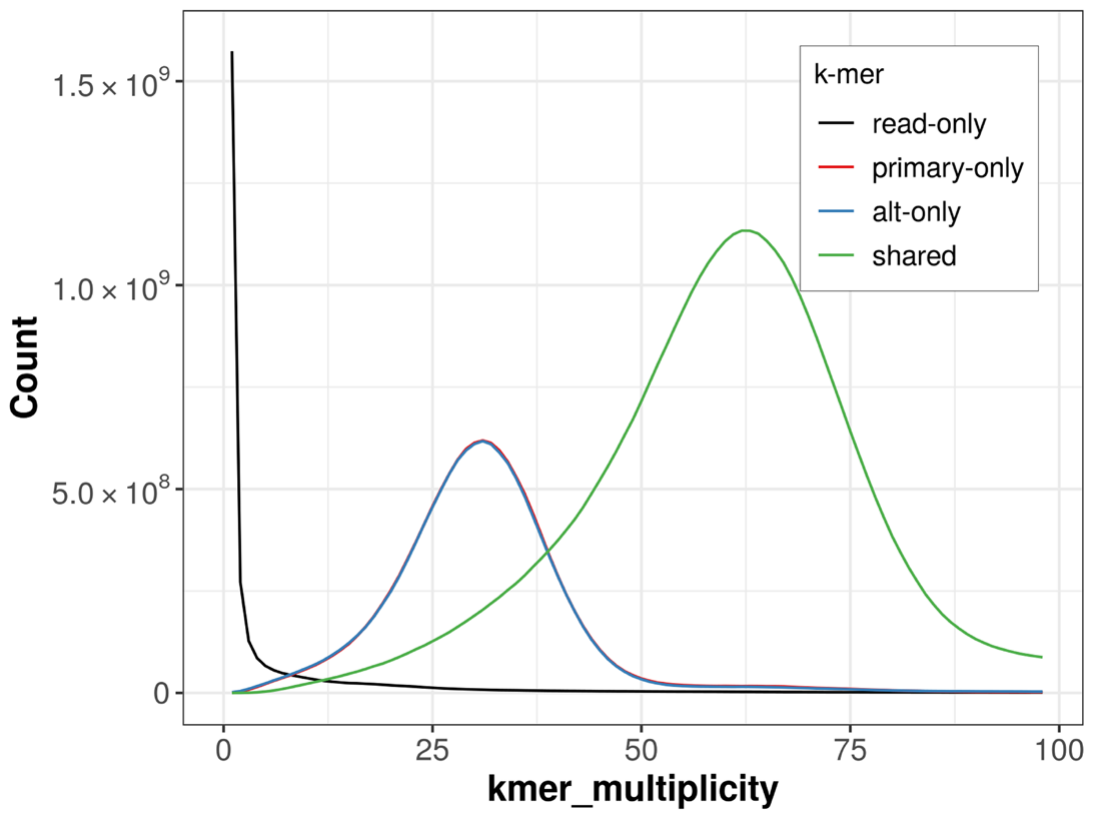
Evaluation of *k*-mer completeness using MerquryFK. This plot illustrates the recovery of *k*‐mers from the original read data in the final assemblies. The horizontal axis represents *k*‐mer multiplicity, and the vertical axis shows the number of *k*‐mers. The black curve represents *k*‐mers that appear in the reads but are not assembled. The green curve corresponds to *k*‐mers shared by both haplotypes, and the red and blue curves show *k*‐mers found only in one of the haplotypes.

The combined primary and alternate assemblies achieve an estimated QV of 56.2. The *k*-mer completeness is 72.36% for the primary assembly, 72.27% for the alternate haplotype, and 98.20% for the combined assemblies ( Figure 4).

**Figure 5.**
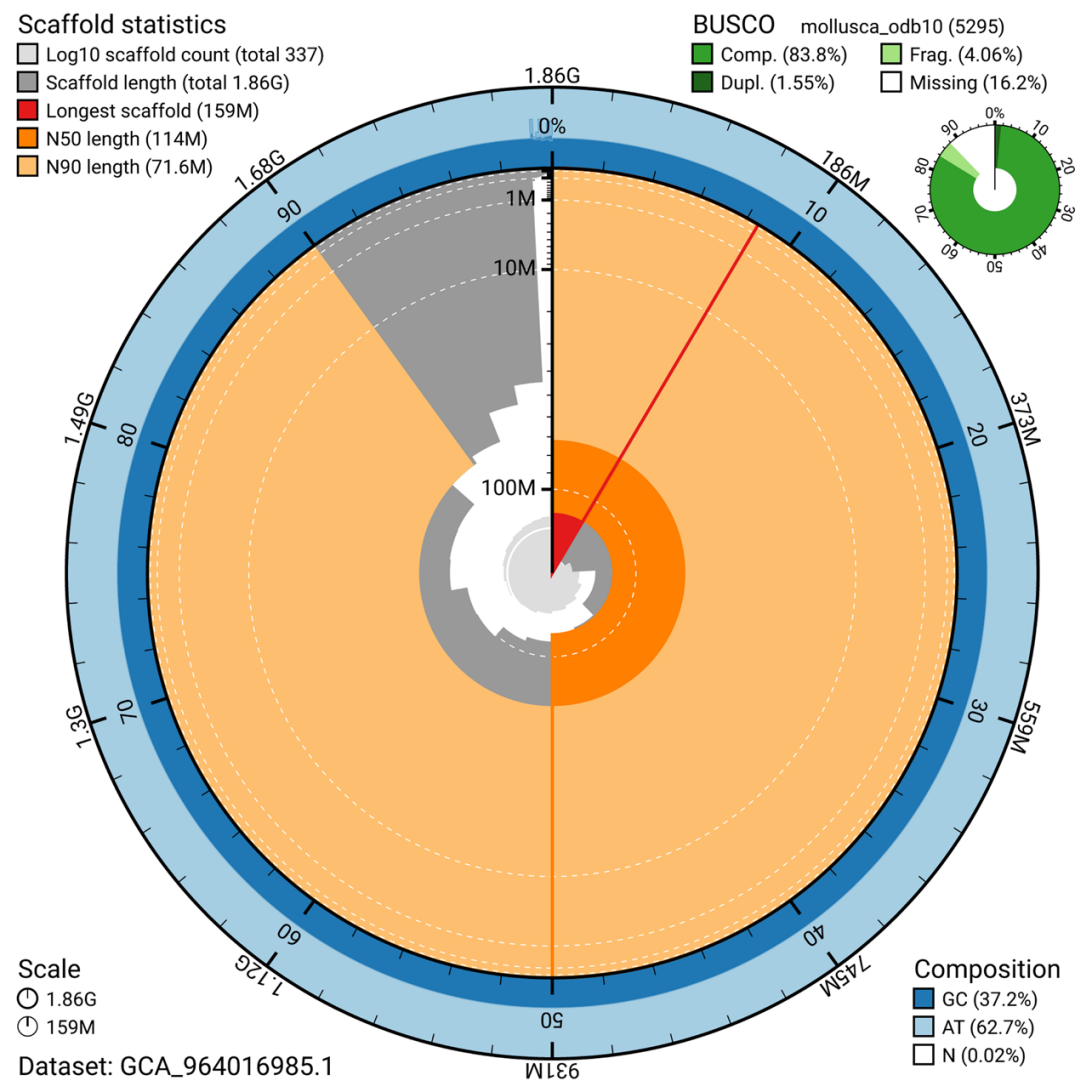
Assembly metrics for xbAnoAlba1.1. The BlobToolKit snail plot provides an overview of assembly metrics and BUSCO gene completeness. The circumference represents the length of the whole genome sequence, and the main plot is divided into 1 000 bins around the circumference. The outermost blue tracks display the distribution of GC, AT, and N percentages across the bins. Scaffolds are arranged clockwise from longest to shortest and are depicted in dark grey. The longest scaffold is indicated by the red arc, and the deeper orange and pale orange arcs represent the N50 and N90 lengths. A light grey spiral at the centre shows the cumulative scaffold count on a logarithmic scale. A summary of complete, fragmented, duplicated, and missing BUSCO genes in the mollusca_odb10 set is presented at the top right. An interactive version of this figure can be accessed on the BlobToolKit viewer.

**Figure 6.**
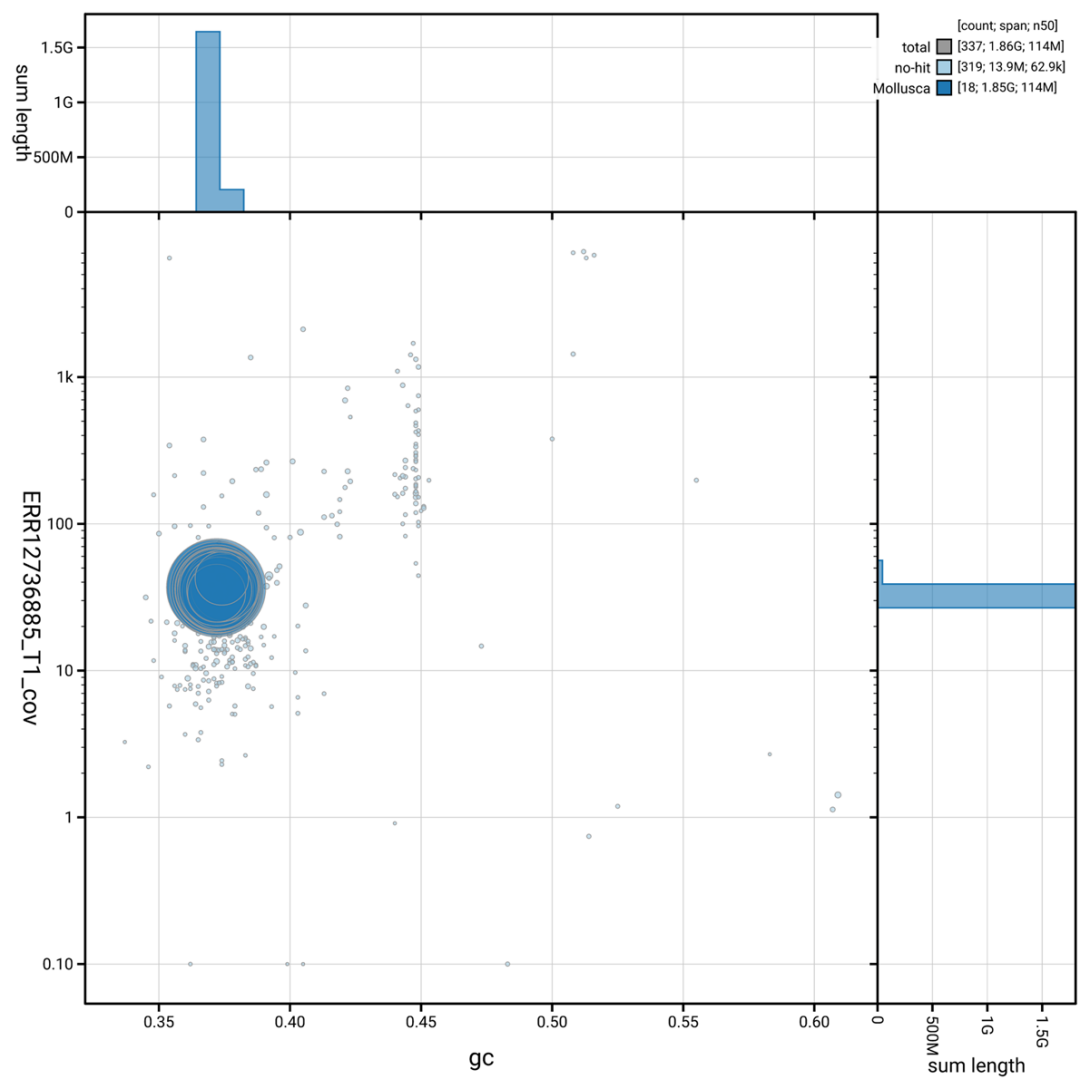
BlobToolKit GC-coverage plot for xbAnoAlba1.1. Blob plot showing sequence coverage (vertical axis) and GC content (horizontal axis). The circles represent scaffolds, with the size proportional to scaffold length and the colour representing phylum membership. The histograms along the axes display the total length of sequences distributed across different levels of coverage and GC content. An interactive version of this figure is available on the BlobToolKit viewer.

BUSCO v.5.5.0 analysis using the mollusca_odb10 reference set ( *n* = 5 295) identified 83.8% of the expected gene set (single = 82.2%, duplicated = 1.5%). The snail plot in Figure 5 summarises the scaffold length distribution and other assembly statistics for the primary assembly. The blob plot in Figure 6 shows the distribution of scaffolds by GC proportion and coverage.

Table 4 lists the assembly metric benchmarks adapted from Rhie *et al.* (2021) and the Earth BioGenome Project Report on Assembly Standards September 2024. The EBP metric, calculated for the primary assembly, is **6.C.Q55**, meeting the recommended reference standard.

**Table 4.** Earth Biogenome Project summary metrics for the *Anodontia alba* assembly.

Measure	Value	Benchmark
EBP summary (primary)	6.C.Q55	6.C.Q40
Contig N50 length	1.84 Mb	≥ 1 Mb
Scaffold N50 length	114.10 Mb	= chromosome N50
Consensus quality (QV)	Primary: 55.6; alternate: 56.8; combined: 56.2	≥ 40
*k*-mer completeness	Primary: 72.36%; alternate: 72.27%; combined: 98.20%	≥ 95%
BUSCO	C:83.8% [S:82.2%; D:1.5%]; F:4.1%; M:12.2%; n:5 295	S > 90%; D < 5%
Percentage of assembly assigned to chromosomes	99.28%	≥ 90%

**Notes:** EBP summary uses log10(Contig N50); chromosome-level (C) or log10(Scaffold N50); Q (Merqury QV). BUSCO: C=complete; S=single-copy; D=duplicated; F=fragmented; M=missing; n=orthologues

### Genome annotation report

The *Anodontia alba* genome assembly (GCA_964016985.1) was annotated by Ensembl at the European Bioinformatics Institute (EBI). This annotation includes 31 170 transcribed mRNAs from 12 083 protein-coding and 11 014 non-coding genes. The average transcript length is 19 295.35 bp, with an average of 1.35 coding transcripts per gene and 5.33 exons per transcript. For further information about the annotation, please refer to the Ensembl annotation page.

## Metagenome report

We recovered four bins from the metagenome assembly, of which three met the criteria for MAGs, including one fully circularised genomes. The recovered bins represented 2 bacterial phyla, with genome sizes ranging from 3.32 to 6.66 Mbp (mean: 4.88 ± 1.21 Mbp). Mean completeness was 90.0% (± 16.1%) with 0.6% (± 0.7%) contamination. Table 5 summarises the taxa and quality of the metagenome bins.

**Table 5.** Quality metrics and taxonomic assignments of the binned metagenomes.

NCBI taxon	Taxid	GTDB taxonomy	Quality	Size (bp)	Contigs	Circular	Mean coverage	Completeness (%)	Contamination (%)
*Pararhizobium* sp.	1977563	Alphaproteobacteria	Medium	3 321 067	136	No	2.71	62.16	0.15
*Candidatus* Thiodiazotropha sp.	2847306	Gammaproteobacteria	High	4 408 143	1	Yes	8 128.05	99.39	1.76
*Rhodococcus* *qingshengii*	334542	Actinomycetes	High	6 655 471	9	No	8.13	99.18	0
*Mycobacterium* sp.	1785	Actinomycetes	High	5 155 284	2	No	18.07	99.17	0.40

Software tool versions and sources are given in Table 6.

**Table 6.** Software versions and sources.

Software	Version	Source
**BEDTools**	2.30.0	https://github.com/arq5x/bedtools2
**bin3C**	0.3.3	https://github.com/cerebis/bin3C
**BLAST**	2.14.0	ftp://ftp.ncbi.nlm.nih.gov/blast/executables/blast+/
**BlobToolKit**	4.3.9	https://github.com/blobtoolkit/blobtoolkit
**BUSCO**	5.5.0	https://gitlab.com/ezlab/busco
**bwa-mem2**	2.2.1	https://github.com/bwa-mem2/bwa-mem2
**checkM**	2015-01-16	https://ecogenomics.github.io/CheckM/
**Cooler**	0.8.11	https://github.com/open2c/cooler
**DIAMOND**	2.1.8	https://github.com/bbuchfink/diamond
**dRep**	3.4.0	https://github.com/MrOlm/drep
**fasta_windows**	0.2.4	https://github.com/tolkit/fasta_windows
**FastK**	1.1	https://github.com/thegenemyers/FASTK
**Gfastats**	1.3.6	https://github.com/vgl-hub/gfastats
**GenomeScope2.0**	2.0.1	https://github.com/tbenavi1/genomescope2.0
**GTDB-Tk**	1.2.1	https://github.com/Ecogenomics/GTDBTk
**Hifiasm**	0.19.8-r603	https://github.com/chhylp123/hifiasm
**HiGlass**	1.13.4	https://github.com/higlass/higlass
**MAGScoT**	1.0.0	https://github.com/ikmb/MAGScoT
**MaxBin**	2.2.7	https://sourceforge.net/projects/maxbin/
**MerquryFK**	1.1.2	https://github.com/thegenemyers/MERQURY.FK
**MetaBAT2**	2.15-15-gd6ea400	https://bitbucket.org/berkeleylab/metabat
**metaMDBG**	Pre-release	https://github.com/GaetanBenoitDev/metaMDBG
**metaTOR**	Pre-release	https://github.com/koszullab/metaTOR
**Minimap2**	2.24-r1122	https://github.com/lh3/minimap2
**MultiQC**	1.14; 1.17 and 1.18	https://github.com/MultiQC/MultiQC
**Nextflow**	23.04.1	https://github.com/nextflow-io/nextflow
**Oatk**	0	https://github.com/c-zhou/oatk
**PretextSnapshot**	0.0.5	https://github.com/sanger-tol/PretextSnapshot
**PretextView**	1.0.3	https://github.com/sanger-tol/PretextView
**Prokka**	1.14.5	https://github.com/tseemann/prokka
**Seqtk**	1.3	https://github.com/lh3/seqtk
**Singularity**	3.9.0	https://github.com/sylabs/singularity
**sanger-tol/ascc**	0.1.0	https://github.com/sanger-tol/ascc
**sanger-tol/blobtoolkit**	0.4.0	https://github.com/sanger-tol/blobtoolkit
**sanger-tol/curationpretext**	1.4.2	https://github.com/sanger-tol/curationpretext
**TreeVal**	1.4.0	https://github.com/sanger-tol/treeval
**YaHS**	1.1a.2	https://github.com/c-zhou/yahs

### Wellcome Sanger Institute – Legal and Governance

The materials that have contributed to this genome note have been supplied by a Tree of Life collaborator. The Wellcome Sanger Institute employs a process whereby due diligence is carried out proportionate to the nature of the materials themselves, and the circumstances under which they have been/are to be collected and provided for use. The purpose of this is to address and mitigate any potential legal and/or ethical implications of receipt and use of the materials as part of the research project, and to ensure that in doing so we align with best practice wherever possible.

The overarching areas of consideration are:

- Ethical review of provenance and sourcing of the material
- Legality of collection, transfer and use (national and international)

Each transfer of samples is undertaken according to a Research Collaboration Agreement or Material Transfer Agreement entered into by the Tree of Life collaborator, Genome Research Limited (operating as the Wellcome Sanger Institute) and in some circumstances other Tree of Life collaborators.

## Data Availability

European Nucleotide Archive: Anodontia alba. Accession number PRJEB73659. The genome sequence is released openly for reuse. The *Anodontia alba* genome sequencing initiative is part of the Aquatic Symbiosis Genomics Project (PRJEB43743) and the Sanger Institute Tree of Life Programme (PRJEB43745). All raw sequence data and the assembly have been deposited in INSDC databases. Raw data and assembly accession identifiers are reported in Table 1 and Table 2. Production code used in genome assembly at the WSI Tree of Life is available at https://github.com/sanger-tol. Table 6 lists software versions used in this study.

## Author information

Contributors are listed at the following links:

- Members of the Wellcome Sanger Institute Tree of Life Management, Samples and Laboratory team
- Members of Wellcome Sanger Institute Scientific Operations – Sequencing Operations
- Members of the Wellcome Sanger Institute Tree of Life Core Informatics team
- Members of the EBI Aquatic Symbiosis Genomics Data Portal Team
- The Aquatic Symbiosis Genomics Project leadership
