# Supplementary material for: The chromosomal genome sequence of the buttercup lucine, Anodontia alba Link, 1807 (Lucinida: Lucinidae) and its associated microbial metagenome sequences
Source: Wellcome Open Res. 2026 Feb 17;11:131. [Version 1] doi: 10.12688/wellcomeopenres.25920.1 (PMC13033139; doi:10.12688/wellcomeopenres.25920.1)
Supplement: Peer review history

# Reviewer report: Reviewer response for version 1

**DOI:** 10.21956/wellcomeopenres.28530.r151560

**Published:** 2026-03-28

**Authors:** Craig Wilding

**Affiliations:** 1 University College Dublin, Dublin, Ireland

The authors present a chromosome-level nuclear genome, mitochondrial genome, and four metagenome-assembled genomes (MAGs) from the bivalve *Anodontia alba*.

 The rationale for sequencing this species is clear, and the methods are clearly presented and thus I have only some very minor queries/suggestions:

 Whilst there is explicit mention in the text that gill tissue was used for Hi-C, there is no similar mention in the text (although it is given in Table 1) of which tissues were used for DNA extraction for PacBio or for RNA extraction. It would be helpful to make this clear in the text.

 Hi-C methods: which REs were in the RE mastermix?

 The genome sequence report states "GenomeScope2.0 analysis estimated the haploid genome size at 2 171.11 Mb, with a heterozygosity of 1.99% and repeat content of 55.18% (Figure 2)." However, the legend of Figure 2 appears to show the estimated length to be 2,167,172,647 bp which is different from that reported in the text. This may be my naivety in reading this plot, but is the figure in the text correct?

 The BUSCO score of complete genes (83.8%) seems low for such a complete genome and importantly it does not meet the EBP benchmark (Table 4) - this really does need commenting upon in the Genome Sequence Report.

 Table 5 - make clear which of these 4 metagenome assemblies was the one that did not meet the criteria for a MAG? Please clarify in the text or Table.

Are sufficient details of methods and materials provided to allow replication by others?

Yes

Is the rationale for creating the dataset(s) clearly described?

Yes

Are the datasets clearly presented in a useable and accessible format?

Yes

Are the protocols appropriate and is the work technically sound?

Yes

Reviewer Expertise:

Evolutionary genetics

I confirm that I have read this submission and believe that I have an appropriate level of expertise to confirm that it is of an acceptable scientific standard.

# Reviewer report: Reviewer response for version 1

**DOI:** 10.21956/wellcomeopenres.28530.r151558

**Published:** 2026-03-28

**Authors:** Mohamed Abdelsalam

**Affiliations:** 1 Cairo University, Giza, Egypt

This Data Note presents a high-quality chromosomal-level genome assembly of *Anodontia alba*, a chemosymbiotic lucinid bivalve of considerable ecological and evolutionary importance. The assembly is technically sound, meets Earth BioGenome Project (EBP) reference standards, and the associated metagenome bins, including three high-quality MAGs, are valuable for the chemosymbiosis research community. The manuscript is well-structured and mostly clear. However, several specific scientific, methodological, and typographical concerns require attention before the final accepted version.

 1. Single-Specimen Genome 

 The entire genome and metagenome assembly is derived from a single individual (xbAnoAlba1) collected from a single location (Sammy Creek Landing, FL, USA). While this is standard practice for genome data notes, the manuscript makes no acknowledgment or a Limitations subsection explicitly addressing single-specimen sampling and the implications of the 72.36% primary k-mer completeness figure.

 2. BUSCO Completeness Below Benchmark — Insufficient Discussion

 The BUSCO score is C:83.8% (S:82.2%, D:1.5%), with 12.2% missing genes against the *mollusca_odb10* reference set. This falls below the EBP benchmark of S > 90%. The manuscript notes this value in Table 4 but provides no narrative explanation for the relatively high proportion of missing BUSCO genes. Is the 12.2% missing BUSCO fraction attributable to repeat-rich assembly regions, gene prediction limitations, or genuine gene loss/divergence in *Anodontia alba* compared to the mollusca_odb10 reference? The authors should at minimum discuss which explanation is most likely, even if empirically unresolved.

 3. Symbiont Identification and Metagenome Report — Key Scientific Gap

 The most scientifically significant metagenome bin is *Candidatus* Thiodiazotropha sp. (a gammaproteobacterial thioautotrophic endosymbiont, 99.39% complete, circularised, with 8,128× mean coverage), which is the known intracellular gill symbiont of *A. alba.*

 -Does the *Candidatus* Thiodiazotropha sp. MAG recovered from this Florida specimen encode nitrogen fixation ( *nif*) genes? Given that the Background explicitly mentions this as a variable trait across *A. alba* populations, this is a key scientific question the metagenome data could directly address.

**-**The two *Actinomycetes* bins ( *Rhodococcus qingshengii* and *Mycobacterium* sp.) are unusual residents for a marine bivalve gill. Are these confirmed as genuine gill-associated microbiota, or are they potential laboratory contaminants or environmental bacteria from the sediment? Their ecological significance in the context of *A. alba* biology is not discussed at all.

 4. Specimen Identification Based Solely on Gross Morphology

 The single specimen was *"identified by Diana Chin (Northeastern University) based on gross morphology"*.

 Confirming specimen identity using a molecular barcode (COI or 16S) or cross-referencing the assembled mitochondrial genome sequence (OZ024839.1, 18.48 kb) against GenBank would greatly strengthen taxonomic confidence.

**-**Was the assembled mitochondrial genome compared against existing *Anodontia alba* or congeneric sequences in GenBank or BOLD? This would provide a direct and inexpensive molecular confirmation of species identity using the data already generated.

 5. Typographical and Language Issues

- Table 2, "Span (Mb)": The value is listed as *1 862.85* using a space as a thousands separator, while elsewhere in the text the same number is written as *1,862.85* or *1 862.85 megabases* inconsistently. A single formatting convention should be used throughout.​
- Table 4, k-mer completeness benchmark: The benchmark is stated as "≥ 95%" but the primary assembly achieves only 72.36%. The authors state the combined value is 98.20% and use this to justify meeting the standard, yet the table row shows the primary value prominently. A footnote clarifying that the combined assembly metric is used for EBP benchmarking would prevent confusion.
- Background, sentence: *"Genomes of Lucinisca nassula (Anderson et al., 2025), Ctena decussata (Wilkins et al., 2025) and Indoaustriella scarlatoi (Guo et al., 2025) have recently been published"* — the word "recently" is appropriate but could be more precisely replaced with "in 2025" for temporal clarity.
- Methods, Nucleic acid extraction: The sentence *"The xbAnoAlba1 sample was weighed and triaged to determine the appropriate extraction protocol"* uses the term "triaged" in an unusual non-clinical context. "Assessed" or "evaluated" would be more precise.​

Are sufficient details of methods and materials provided to allow replication by others?

Yes

Is the rationale for creating the dataset(s) clearly described?

Yes

Are the datasets clearly presented in a useable and accessible format?

Yes

Are the protocols appropriate and is the work technically sound?

Yes

Reviewer Expertise:

genetics

I confirm that I have read this submission and believe that I have an appropriate level of expertise to confirm that it is of an acceptable scientific standard.
